# Dispersion-mediated steering of organic adsorbates on a precovered silicon surface

**DOI:** 10.3762/bjoc.14.249

**Published:** 2018-10-26

**Authors:** Lisa Pecher, Sebastian Schmidt, Ralf Tonner

**Affiliations:** 1 Fachbereich Chemie and Material Sciences Center, Philipps-Universität Marburg, Hans-Meerwein-Straße 4, 35032 Marburg, Germany

**Keywords:** bonding analysis, cyclooctyne, density functional theory, dispersion, organic/inorganic interfaces

## Abstract

The chemistry of organic adsorbates on surfaces is often discussed in terms of Pauli repulsion as limiting factor regarding the packing of molecules. Here we show that the attractive part of the van der Waals potential can be similarly decisive. For the semiconductor surface Si(001), an already covalently bonded molecule of cyclooctyne steers a second incoming molecule via dispersion interactions onto the neighbouring adsorption site. This helps in understanding the nonstatistical pattern formation for this surface–adsorbate system and hints toward an inclusion of dispersion attraction as another determining factor for surface adsorption.

## Introduction

The creation of organic/inorganic interfaces is one of the main endeavours in enhancing the application range of modern electronic devices for silicon-based technology [1–2]. One way to achieve this is covalent attachment of bifunctional organic molecules on bare silicon surfaces and subsequent reaction with a second molecule with both reactions being chemoselective (layer-by-layer, LbL, approach) [3–5]. To achieve an interface structure with predictable properties, it is important that the molecules used for the first layer show well-defined surface chemistry without side reactions and lead to densely packed and well-ordered structures.

Cyclooctyne (**1**), the smallest stable cyclic alkyne, on Si(001) is a system where this is the case and it has previously been thoroughly studied by experiment and theory [6–8]. Even though **1** is missing a second functional group necessary for the LbL approach, previous studies have shown that synthetic routes exist for derivatization and that the reactivity of the strained triple bond of **1** with the surface is not affected by the second functional group [4–5,9]. Studying the adsorption behaviour of the parent system **1** thus gives crucial insight that is expected to be transferable to the bifunctional derivatives.

**Figure 1:**
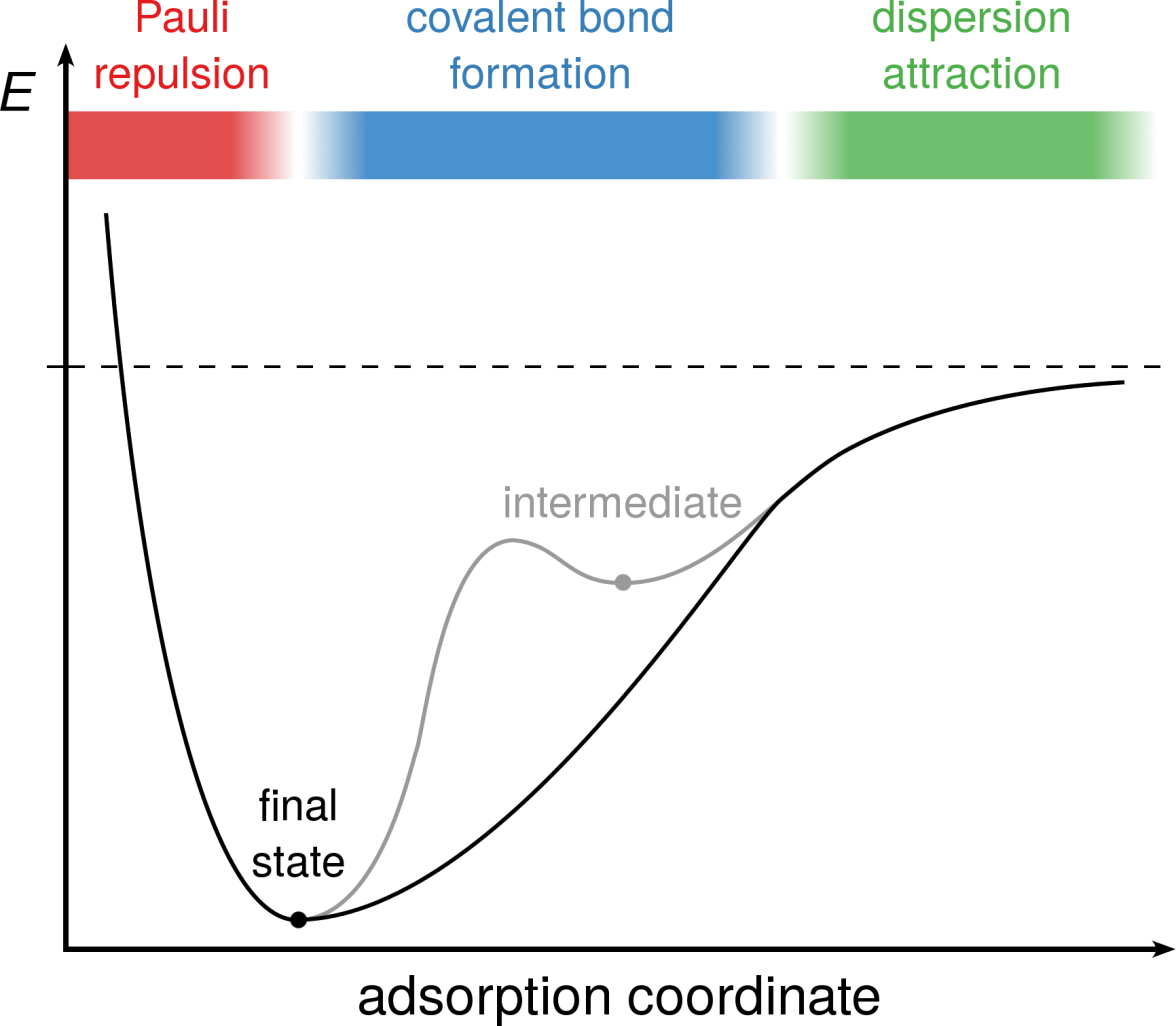
Adsorption energy profile of a direct adsorption (black, e.g., cyclooctyne/Si(001)) in comparison with adsorption via an intermediate (grey, e.g., methanol/Ge(001)). On top, the dominant type of interaction in the different regimes of the profile is given.

The adsorption of a molecule on a surface can proceed either via a direct pathway or via an intermediate species that is crucial for selectivity and the description of adsorption dynamics (Figure 1). The dominant interaction between molecule and surface changes with the distance: For surface–adsorbate distances at which there is no significant orbital overlap but already rather close contact, dispersion attraction dominates since the numerous rather weak interactions add up to a significant stabilization, especially for larger adsorbates and/or polarizable substrates. At shorter distances, covalent bond formation leads to a steeper attractive potential well. The resulting covalently bonded state is usually called chemisorbed [10]. Bonding of intermediate states can be dominated by dispersion or covalent interactions. At very short distances, Pauli repulsion creates the repulsive potential wall.

Experimental studies in combination with Monte Carlo simulations have shown that growth of **1** on Si(001) results in non-statistical formation of chains with an average distance of 1.5 to 2 dimers between adsorbates [6]. For the adsorption of methanol on Ge(001), where a similar behaviour is observed, it was shown that in an intermediate state, interactions with other adsorbed molecules lead to a reduction of the energy barrier for conversion into the final chemisorbed state [11]. This accelerates the adsorption next to occupied sites and leads to the formation of 1D chains, as derived from computations. However, since the adsorption of cyclooctyne on Si(001) is direct or pseudo-direct and does not proceed via an intermediate [5–6,8], this explanation is not applicable. Previously, it was proposed that the occupied sites might “steer” impinging molecules via an attractive adsorption potential close to an already adsorbed molecule [6].

Here, we will show that this steering potential is indeed found and is caused by attractive dispersion interactions. To this end, we investigated the adsorption of a molecule on a pre-covered surface using density functional theory (DFT) approaches with and without dispersion correction terms. In contrast to the above-mentioned intermediate-based selectivity, the steering-type interaction takes place before covalent bonds between molecule and surface are formed. The results show that dispersive interactions can be decisive in building novel organic structures on surfaces by tweaking the potential energy surface.

## Computational Details

All calculations were performed with the Vienna Ab Initio Simulation Package (VASP) [12–15] version 5.3.5 using the PBE functional [16–17], the DFT-D3 dispersion correction [18–19] and the PAW formalism [20–21] with a basis set cutoff of *E*_cutoff_ = 400 eV. Electronic *k* space was sampled using a Γ(221) grid. Some calculations used the DFT-TS scheme for comparison [22]. Self-consistent field (SCF) and structural optimization convergence criteria were set to 10^−6^ eV and 10^−2^ eV·Å^−1^, respectively. Structures were optimized using the Conjugate Gradient algorithm [23] and Gibbs energies were calculated at *T* = 300 K, *p* = 1 bar using an approach described elsewhere [24]. Harmonic vibrational frequencies used in the calculation of Gibbs energies were derived by numerical construction of the Hessian using Cartesian displacements of 0.01 Å from the equilibrium structure. The Si(001) surface was modelled as a six-layer slab in *c*(4 × 2) reconstruction with 4 × 4 atoms per layer. The frozen double layer approximation was applied (i.e., the bottom two layers were not relaxed in structural optimizations) and the bottom layer saturated with hydrogen atoms in tetrahedral arrangement at *d*(Si-H) = 1.480 Å, the experimental equilibrium distance in silane [25]. Cell constants *a* and *b* (in *x* and *y* direction) were set to 15.324 Å, derived from an optimized bulk parameter of 5.418 Å for this computational setup [7], while in *z* direction, a vacuum layer of at least 10 Å was ensured. The bonding energy *E*_bond_ was defined as the energy difference between the relaxed structures of the total system (*E*_tot_) and the isolated molecule (*E*_mol_) and surface (*E*_surf_):


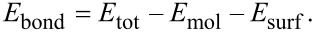


Please note that in case of a precovered surface, *E*_surf_ also includes the already adsorbed molecule, and that surface science convention is the use of the adsorption energy *E*_ads_ with inverse sign convention (*E*_ads_ = −*E*_bond_).

Adsorption energy profiles were calculated by placing the cyclooctyne molecule in an upright orientation (molecular *C*_2_ axis aligned parallel to the *z* axis of the cell), with the triple bond aligned parallel to the *y* axis of the cell, the triple-bond centre located vertically above a lower surface atom (Si_down_) at a height corresponding to a vertical distance between the triple-bond carbon atoms and the uppermost surface atoms (Si_up_) of Δ*z*(Si_up_–C_triple_) = 4 Å. The system was then optimized using the Conjugate Gradient algorithm. In a previous study, we have shown that this approach yields an energy profile that is in qualitative agreement with the true minimum energy path for this system [8].

Potential energy surface scans were performed by displacing a cyclooctyne molecule in *x* and *y* direction while retaining the orientation (equivalent to the starting point of the adsorption energy profile) and a fixed distance Δ*z*(Si_up_–C_triple_) above the surface. The displacement grid was chosen to consist of 20 × 20 equidistant points spanning the whole unit cell, corresponding to a distance of 0.766 Å between individual grid points. Since the system was not optimized at each grid point, this corresponds to a so-called frozen scan. The approach outlined here has delivered accurate results for organic/semiconductor systems in the past [7–9,24].

## Results and Discussion

### Bonding and the adsorption path

**Scheme 1:**
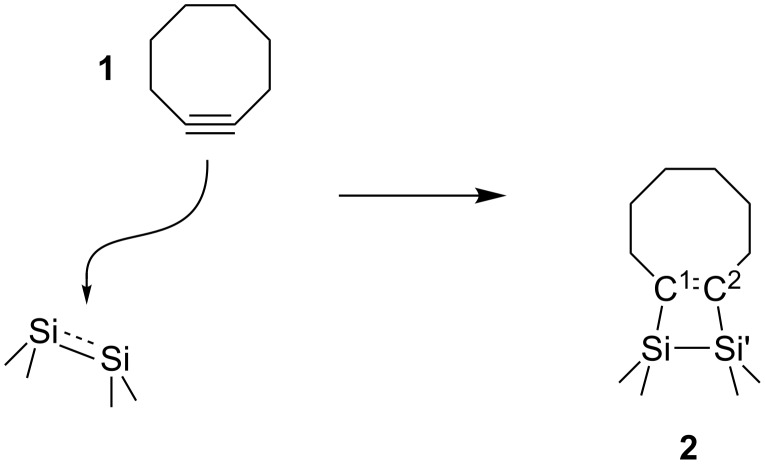
The reaction of cyclooctyne (**1**) with a Si(001) surface dimer, yielding a [2 + 2] cycloadduct **2**.

The reactivity of the Si(001) surface is dominated by Si surface dimers with an electronic structure that is well represented by an electrophilic and a nucleophilic Si atom. The adsorption of a first molecule of **1** on Si(001) is characterized by a direct adsorption path without intermediate structure leading to a strongly covalently bonded [2 + 2] cycloaddition product **2** as summarized in Scheme 1 with ring strain being decisive for the high reactivity of **1** [5–6,8]. Not reflected in the Lewis structure is the tilting of the molecule upon adsorption leading to a chair-like conformer bending over the dimer rows on the surface [7].

**Figure 2:**
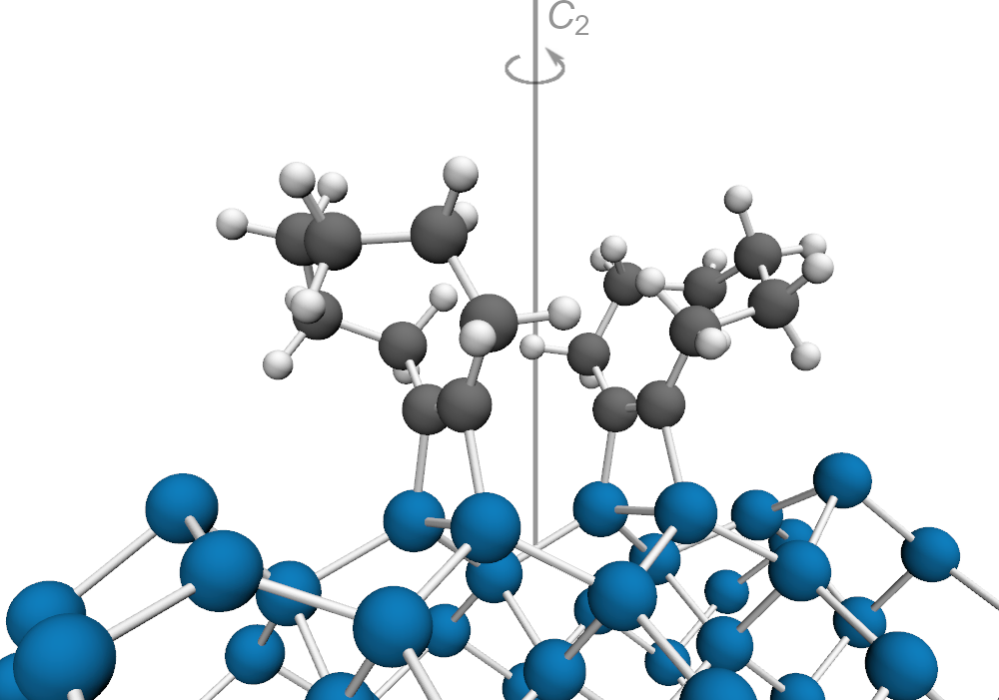
Optimized (PBE-D3/PAW) structure of two molecules of **1** on Si(001) on neighbouring surface dimers (**3**).

Starting from this precovered surface (i.e., decorated with one adsorbate in the unit cell), we now investigate the adsorption of a second molecule of **1** on a neighbouring dimer leading to structure **3** (Figure 2). Although repulsive interactions might be expected for adsorption close to a rather large adsorbate, we find this mode to be the most stable adsorption mode for two molecules of **1** in the unit cell. Due to their conformational flexibility, both molecules **1** and **1′** bend away from each other (Figure 2), thus reducing steric repulsion as further discussed below. An alternative structure where both cyclooctyne molecules bend in the same direction is higher in energy (+4 kJ·mol^−1^), although dispersion attraction is slightly more stabilizing compared to **3** (by 3 kJ·mol^−1^). We will thus focus our discussion on the minimum-energy structure.

Comparison of key structural parameters (Table 1) shows that the C–C as well as the C–Si bond lengths are essentially unaffected by the presence of the second molecule. Interestingly, the energy minimization without symmetry constraints leads to a structure with a local *C*_2_ rotational axis resulting in symmetry-equivalent molecules **1** and **1′**.

**Table 1:** Selected interatomic distances (in Å) of **2** and **3**.^a^

	*d*(C^1^–C^2^)	*d*(C^1^–Si)	*d*(C^2^–Si′)

**2**^b^	1.368	1.916	1.900
**3**^c^	1.368	1.915	1.904

^a^See Scheme 1 for nomenclature; ^b^values taken from [7]; ^c^the two molecules are symmetry-equivalent.

Energies and Gibbs energies of adsorption were previously found to support the notion of strong covalent bonding for the [2 + 2] cycloaddition of **1** on the silicon surface [7]. The adsorption energy for a second molecule on the precovered surface is now found to be even slightly larger by 11 kJ·mol^−1^ (Table 2). This is surprising at first since the presence of a rather bulky adsorbate on the surface should lead to a blocking of neighbouring sites by Pauli repulsion. As we will see later, this is indeed the case for one of the neighbouring dimers. But due to the tilting of the first molecule of **1**, the second adsorbate is not hindered by repulsive interactions. The electronic contribution to the adsorption energy (*E*_bond_(PBE) in Table 2) is indeed unchanged. On the contrary, the dispersion contribution shows an increase for the second adsorbate (−55 kJ·mol^−1^ vs −47 kJ·mol^−1^ for adsorption on the clean surface), which is the main cause for the slightly larger bonding energy.

**Table 2:** Adsorption energies (in kJ·mol^−1^) of **1** on a precovered Si(001) surface leading to **3** compared with the corresponding values for adsorption on a clean surface leading to **2**.

	clean surface^a^	precovered surface

*E*_bond_(PBE)^b^	−261	−264
*E*_bond_(D3)^b^	−47	−55
*E*_bond_(PBE-D3)	−308	−319
*G*_bond_(PBE-D3)	–238	−249

^a^Values taken from [7]; ^b^electronic (PBE) and dispersive (DFT-D3) contributions adding up to *E*_bond_, derived from the PBE-D3 structure.

Energy decomposition analysis for both structures (Table 3) confirms that indeed Pauli repulsion is virtually the same for adsorption on the clean (Δ*E*_Pauli_ = 1468 kJ·mol^−1^) and precovered surface (Δ*E*_Pauli_ = 1467 kJ·mol^−1^) while small changes in electrostatic (Δ*E*_elstat_) and orbital (Δ*E*_orb_) contributions compensate each other. This leaves the increase in dispersion interaction by 8 kJ·mol^−1^ for the precovered surface as the major, albeit small, contribution to the slightly larger interaction energy thus confirming the finding above. Thus, the changes in the pEDA energy terms are rather small but the most important observation is that Pauli repulsion does not significantly rise as is often found for the adsorption of molecules on precovered surfaces [24].

**Table 3:** Energy decomposition analysis (pEDA) results (PBE-D3/TZ2P) for the adsorption of **1** on a clean and precovered Si(001) surface. All values in kJ·mol^−1^.

	clean surface^a^		precovered surface	

Δ*E*_int_	−658		−668	
Δ*E*_int_(disp)	−43 (7%)		−51 (8%)	
Δ*E*_int_(elec)	−615 (93%)		−616 (92%)	

Δ*E*_Pauli_	1468		1467	
Δ*E*_elstat_	−936 (45%)		−949 (46%)	
Δ*E*_orb_	–1148 (55%)		−1134 (54%)	

Δ*E*_prep_(mol.)	313		312	
Δ*E*_prep_(surf.)	26		30	
				
*E*_bond_^b^	−319 (−308)		−325 (−319)	

^a^Values taken from [7]; ^b^PAW values (in parentheses) given for comparison.

**Figure 3:**
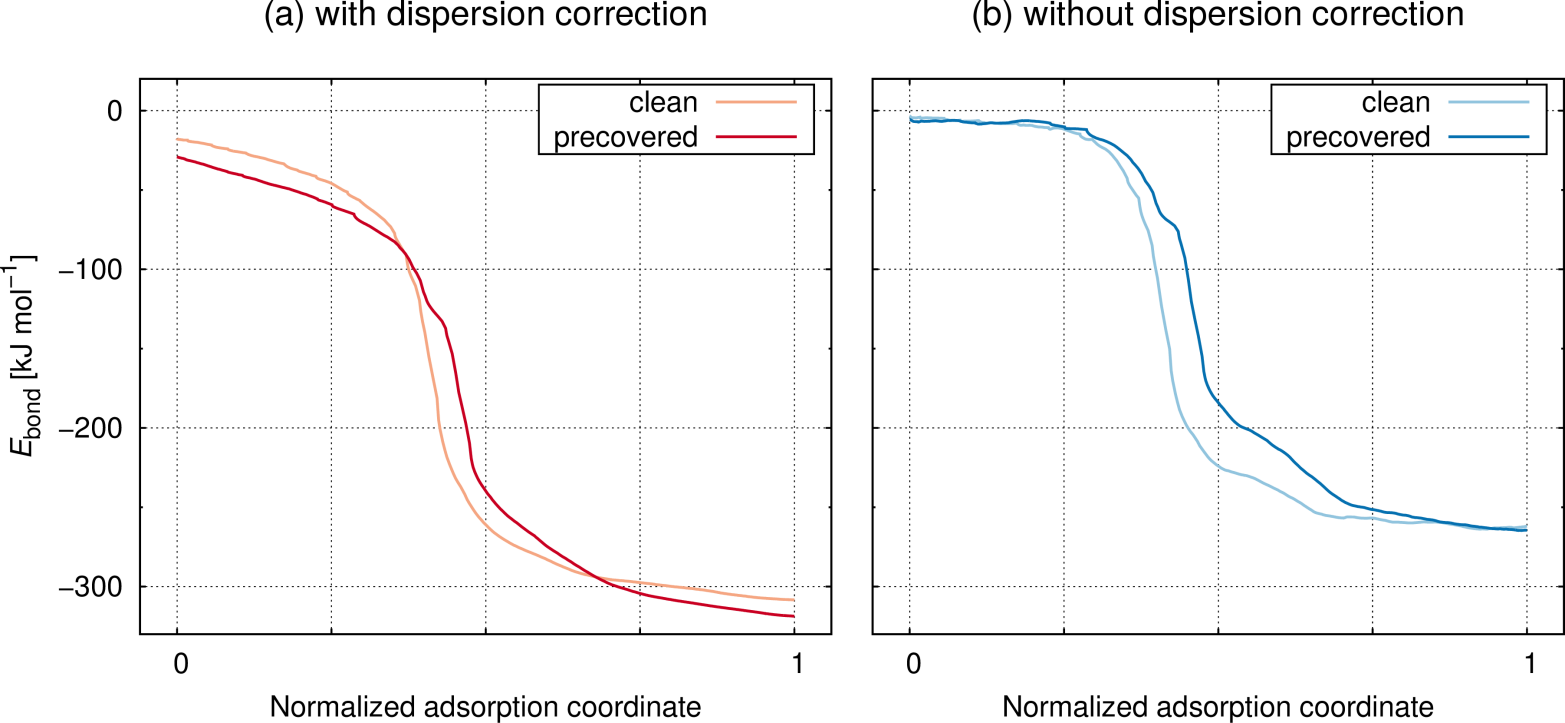
Energy profile of the adsorption pathway depicted in Scheme 1 on the clean and precovered Si(001) surface computed with (a) PBE-D3 and (b) PBE.

The bonding in the covalent [2 + 2] cycloaddition product (i.e., the final state of adsorption) is thus very similar for clean and precovered surfaces. But the reaction path leading to this state might still be qualitatively changed by the presence of a molecule **1** on the surface. The comparison of optimized adsorption paths for clean and precovered surface in Figure 3 shows that this is not the case. In agreement with experimental observation and our previous findings, a direct pathway is observed for the adsorption of **1** on the silicon surface without an intermediate that would show up as stationary point in the energy profile [5–7]. The only difference is found in the adsorption paths when dispersion corrections are included in the computation (Figure 3a).The curve is rather constantly shifted by 5–10 kJ·mol^−1^ towards more negative bonding energies *E*_bond_ in case of the precovered surface. This is not found in the computation that omits dispersion forces (Figure 3b). Thus, dispersion interactions not only stabilize product **3** but act along the whole adsorption path of **1** onto Si(001). This leads us to a comprehensive investigation of the potential energy surface of adsorption.

### The potential energy surface

**Figure 4:**
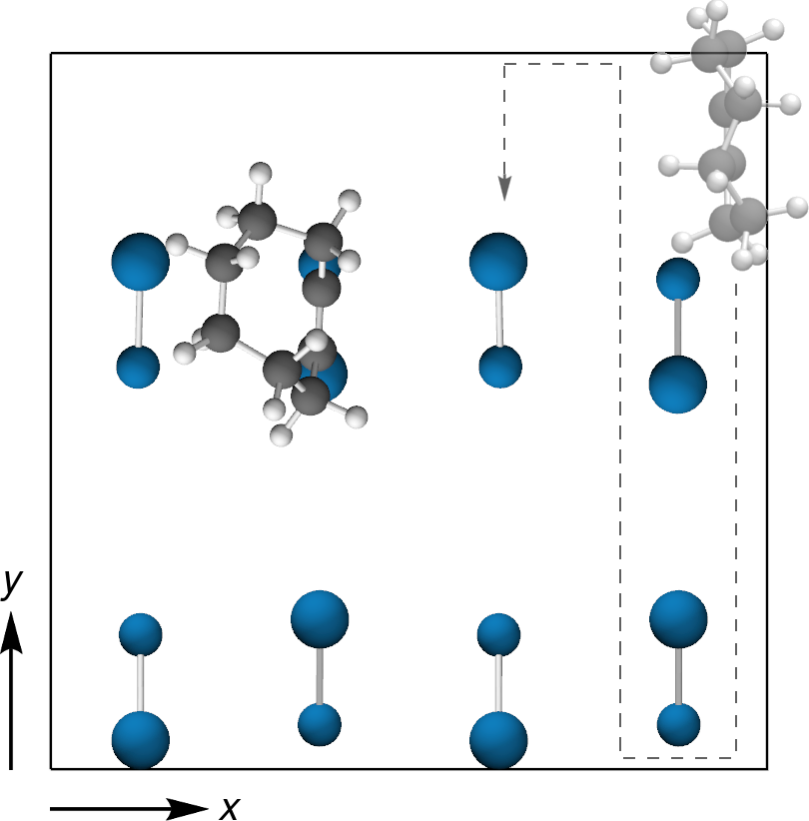
Si(001) Surface precovered with one adsorbate **1** and unit cell used in the PES scans (orientation of second adsorbate and scan path indicated) shown in Figure 5.

Since both the product and the pathway are influenced by dispersion interactions, the question arises if these forces can tweak the potential energy surface (PES) in a way to steer the second adsorbate onto a certain position on the surface. We investigated this by conducting rigid PES scans on the clean and precovered surface by systematically placing **1** on a grid of possible positions at a fixed distance to the surface (Figure 4). We considered different adsorption heights and orientations of **1** (see “Computational Details” and Supporting Information File 1).

**Figure 5:**
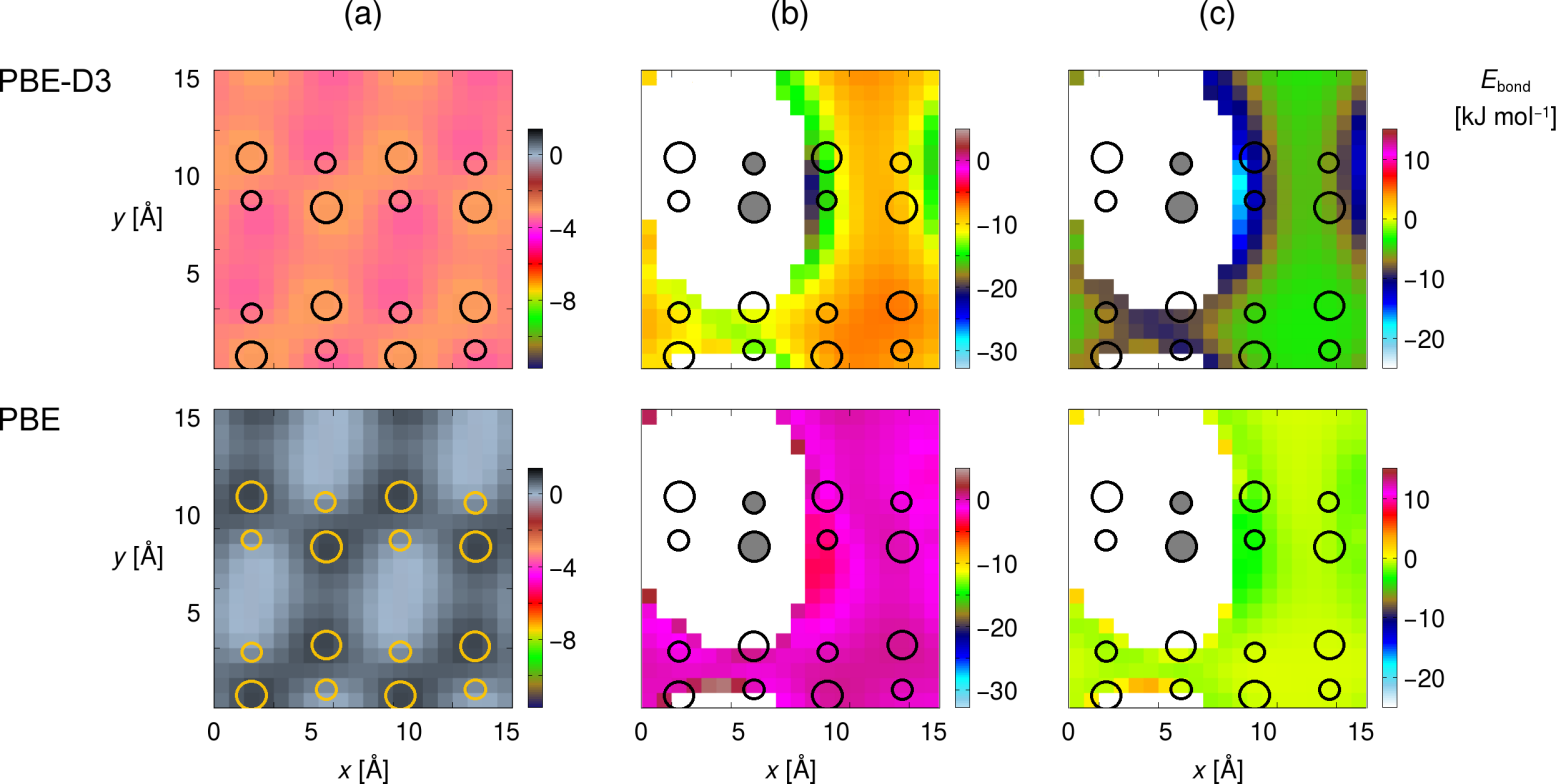
Frozen PES scans of **1** along path indicated in Figure 4 (*C*_2_ axis parallel to *z*, C≡C parallel to *y*, Δ*z*(Si_up_–C_triple_) = 5 Å) on (a) the clean and (b) the precovered surface, and (c) the difference between (a) and (b). Circles denote surface atoms, grey shading denotes the occupied site. White region: no values given due to highly repulsive interactions or overlapping molecules.

The most interesting data set is found for the case where the triple bond of **1** points toward the surface at an adsorption height of Δ*z*(Si_up_–C_triple_) = 5 Å (Figure 5). For the clean surface, the PES is essentially featureless (Figure 5a) and only minor effects (stabilization by less than 5 kJ·mol^−1^) are found by including dispersion in the computation. If one molecule of **1** is already present at the surface, parts of the surface are inaccessible due to strong Pauli repulsion and overlapping molecules. This is indicated by the white areas around the first adsorbate in Figure 5b. Now, significant differences can be found between the PES scan with (top panels) and without (bottom panels) considering dispersion effects. For the computations without dispersion correction, the PES is again rather featureless and the interaction between adsorbate and surface is very weak. This can be seen in the difference plot between the PES of the clean and precovered surface in Figure 5c. Only weak preference for the surface dimer adjacent to the already adsorbed molecule is found (less than 5 kJ·mol^−1^), which can be attributed to weak electrostatic attraction between the two molecules.

The picture changes completely when dispersion attraction is considered. The PBE-D3 computations show a pronounced feature in the PES scan on the precovered surface with a strong energetic preference for adsorption on the surface dimer next to the first adsorbate. The stabilization can be seen in the difference to the PES of the clean surface (Figure 5c) and amounts to ca. 20 kJ·mol^−1^ out of a total molecule–surface attraction of ≤25 kJ·mol^−1^. Notably, the tilting of **1** in structure **2** (Figure 4) thus leads to a blocking of one adjacent dimer in *x* direction, but an adsorption preference on the other adjacent dimer leading to structure **3** (Figure 2).

This preference is most pronounced for an adsorption height of 5 Å shown here but is also found for vertical distances of 3 and 7 Å to the surface (Figure S1, Supporting Information File 1). It is also not an artifact of the dispersion correction method chosen (DFT-D3) since a scan with a second method (DFT-TS) leads to the same picture with only slight numeric differences (Figure S2, Supporting Information File 1).

The double-adsorption structure **3** will now lead to a blocking of two dimers and thus result on average in a distance of two dimers between adsorbates. As was shown before, **1** can also adsorb in the twist-boat conformation leading to an arrangement of three molecules on three consecutive dimers [7]. The resulting coverage is thus in agreement with the coverages derived from analysis of the experimental structure [6].

## Conclusion

We have shown that dispersion effects are not only important for the thermodynamic stability of molecule–adsorbate complexes but they also crucially influence the adsorption path. While Pauli repulsion is often discussed as important effect for determining surface adsorption, the attractive part of the van der Waals potential can be of similar importance. For the system cyclooctyne on Si(001), attractive dispersion interactions lead to a preferred adsorption of a second molecule in the neighbourhood of a first adsorbate – an arrangement that is often excluded due to Pauli repulsion arguments. Experimental observation of nonstatistical chain formation can thus be explained. Especially for larger adsorbates, these attractive interactions are expected to play an important role in determining the surface arrangement of molecules and might thus be even used for designing patterned surfaces. To this end, ab initio modelling that accounts for dispersion interactions plays an important role.

## Supporting Information

The supporting information shows PES scans comparing DFT-D3 and DFT-TS, scans at different adsorption heights as well as Cartesian coordinates and total energies for the equilibrium structures presented.

File 1 Additional calculational data.
