# Supplementary material for: Dispersion-mediated steering of organic adsorbates on a precovered silicon surface
Source: Beilstein J Org Chem. 2018 Oct 26;14:2715–21. doi: 10.3762/bjoc.14.249 (PMC6204779; doi:10.3762/bjoc.14.249)
Supplement: File 1 — Additional calculational data. [file Beilstein_J_Org_Chem-14-2715-s001.pdf]

Supporting Information

for

Dispersion-mediated steering of organic adsorbates on a precovered silicon surface

Lisa Pecher, Sebastian Schmidt and Ralf Tonner*

Address: Fachbereich Chemie and Materials Science Center, Philipps-Universität Marburg, Hans-Meerwein-Straße 4, 35032 Marburg, Germany

Email: Ralf Tonner* - tonner@chemie.uni-marburg.de

* Corresponding author

Additional calculational data

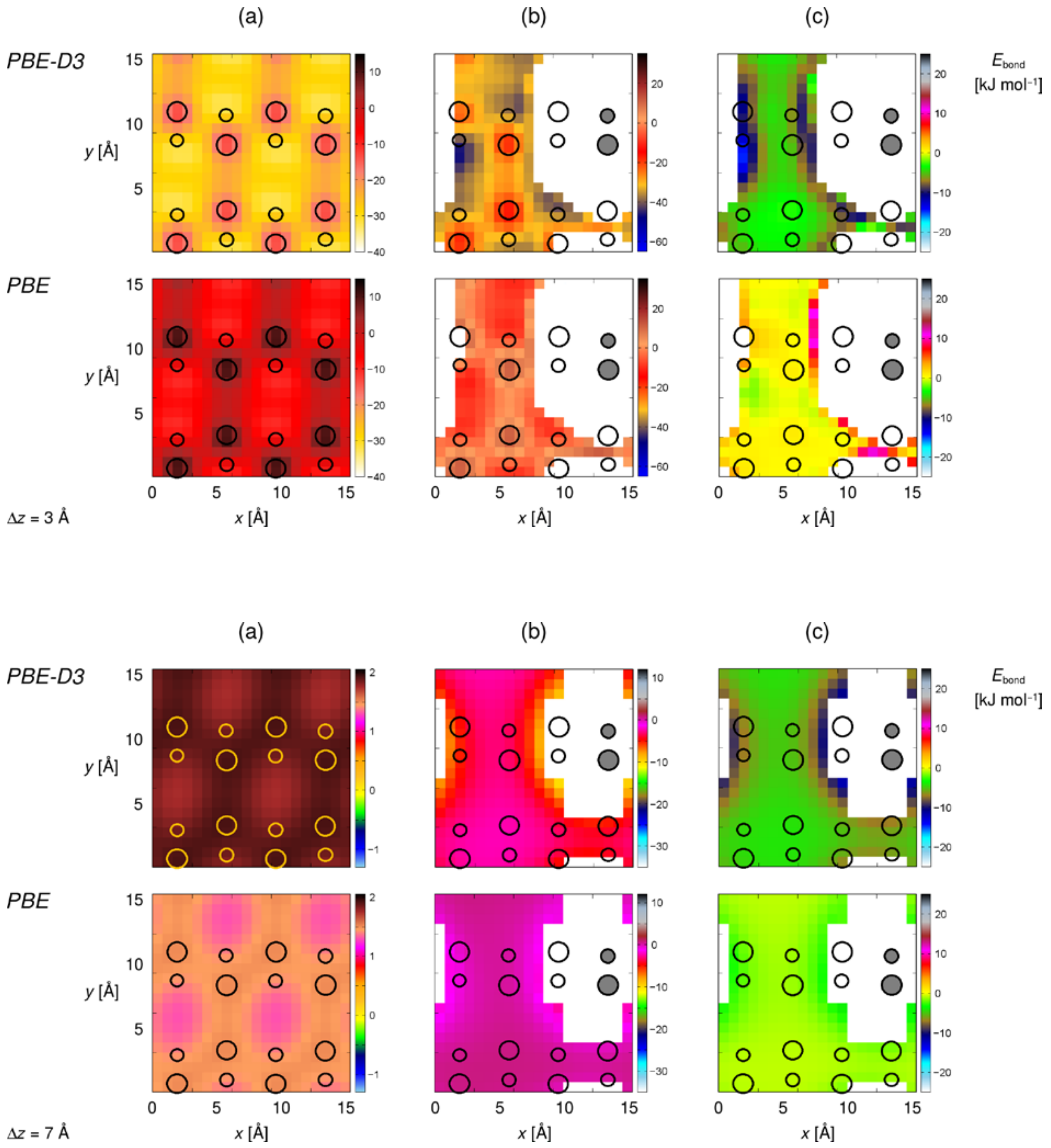

Figure S1: Frozen PES scans of **1** (C_2 axis ∥ z, C≡C ∥ y, Δz(Si$_{up}$-C$_{triple}$) = 3 (top) and 7 Å (bottom)) on the (a) clean, (b) precovered Si(001) surface and (c) the difference between (a) and (b). Circles denote surface atoms, grey shading denotes the occupied site. White region: No values given due to highly repulsive interactions or overlapping molecules.

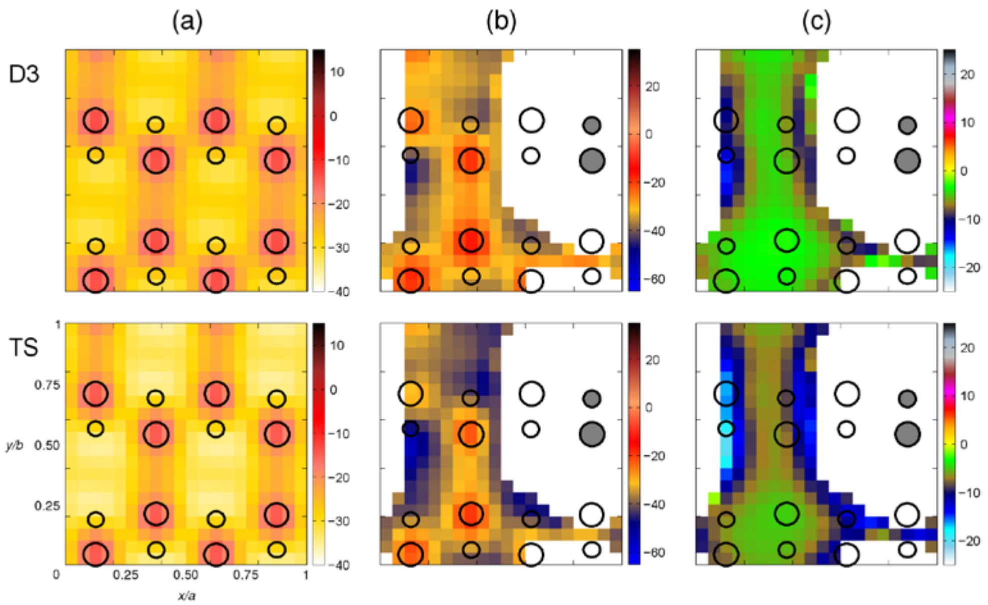

Figure S2: Frozen PES scans of **1** (C_2 axis ∥ z, C≡C ∥ y, $\Delta z(Si_{up}-C_{triple})$ = 3 Å) on the (a) clean, (b) precovered Si(001) surface and (c) the difference between (a) and (b) with PBE-D3 (top panels) and PBE-TS (bottom panels). Circles denote surface atoms, grey shading denotes the occupied site. White region: No values given due to highly repulsive interactions or overlapping molecules.

Cartesian Coordinates and Total Energies

Fractional coordinates (in VASP format) and total energies (in eV) including constraints used in optimization (F/T indicators) for the optimized structures presented in the manuscript.

Cyclooctyne Molecule **1**

```
E = -115.01046 eV
   1.00000000000000
    15.3244190216000007    0.0000000000000000    0.0000000000000000
     0.0000000000000000   15.3244190216000007    0.0000000000000000
     0.0000000000000000    0.0000000000000000   21.6720008849999992
   H    C
    12     8
Direct
  0.1163292027615839  0.6075905129068531  0.6489572235432988
  0.0432877432463101  0.6285424276006779  0.5883106440942782
  0.1499959959152122  0.4908745064372297  0.5880627771758000
  0.2288209192430060  0.5660420583681045  0.5611408236228854
  0.0578964740925230  0.5117902870342448  0.4973925222925502
```

0.1904054750395119	0.8220361050279124	0.4822764132445130
0.1561410273173252	0.7463691841605851	0.6429950518866505
0.2195764077142996	0.7174582486771399	0.5786903955150677
0.0306393927154573	0.7771647276382865	0.5621253712003664
0.0791274400424058	0.8380471554892495	0.4638629576123918
0.1128068282114540	0.8550615537008071	0.5778717888148819
0.1657336841321140	0.4932807017369711	0.4714541689074920
0.1222223218395584	0.7051062349559629	0.4653092188627923
0.1133722423679444	0.6269314805172215	0.6000332349693096
0.1580897035959765	0.5531165957501614	0.5631557335818798
0.1244806956188427	0.7941845194493524	0.4882726092733662
0.1011920815474809	0.7899406237531252	0.5578110578395723
0.1516884602251025	0.7208810184923351	0.5955587832743490
0.1241332066627265	0.5401503162320509	0.4964036956098781
0.1221637132009548	0.6259437125635898	0.4678541608159250

Si(001) Slab

E = -627.24130 eV

1.00000000000000

15.3244190216000007	0.0000000000000000	0.0000000000000000
0.0000000000000000	15.3244190216000007	0.0000000000000000
0.0000000000000000	0.0000000000000000	21.6720008849999992

Si H

96 32

Selective dynamics

Direct

0.0000000000000000	0.0000000000000000	0.0438344851055064	F	F	F
0.5000000319229088	0.0000000000000000	0.0438344851055064	F	F	F
0.0000000000000000	0.2500000159614544	0.0438344851055064	F	F	F
0.5000000319229088	0.2500000159614544	0.0438344851055064	F	F	F
0.2500000159614544	0.0000000000000000	0.0438344851055064	F	F	F
0.7500000478843631	0.0000000000000000	0.0438344851055064	F	F	F
0.0000000000000000	0.5000000319229088	0.0438344851055064	F	F	F
0.5000000319229088	0.5000000319229088	0.0438344851055064	F	F	F
0.2500000159614544	0.2500000159614544	0.0438344851055064	F	F	F
0.7500000478843631	0.2500000159614544	0.0438344851055064	F	F	F
0.0000000000000000	0.7500000478843631	0.0438344851055064	F	F	F
0.5000000319229088	0.7500000478843631	0.0438344851055064	F	F	F
0.2500000159614544	0.5000000319229088	0.0438344851055064	F	F	F
0.7500000478843631	0.5000000319229088	0.0438344851055064	F	F	F
0.2500000159614544	0.7500000478843631	0.0438344851055064	F	F	F
0.7500000478843631	0.7500000478843631	0.0438344851055064	F	F	F
0.1249999753530631	0.0000000000000000	0.1063344825532511	F	F	F
0.6250000072759718	0.0000000000000000	0.1063344825532511	F	F	F
0.1249999753530631	0.2500000159614544	0.1063344825532511	F	F	F
0.6250000072759718	0.2500000159614544	0.1063344825532511	F	F	F
0.3749999913145174	0.0000000000000000	0.1063344825532511	F	F	F
0.8750000232374191	0.0000000000000000	0.1063344825532511	F	F	F
0.1249999753530631	0.5000000319229088	0.1063344825532511	F	F	F
0.6250000072759718	0.5000000319229088	0.1063344825532511	F	F	F
0.3749999913145174	0.2500000159614544	0.1063344825532511	F	F	F
0.8750000232374191	0.2500000159614544	0.1063344825532511	F	F	F
0.1249999753530631	0.7500000478843631	0.1063344825532511	F	F	F
0.6250000072759718	0.7500000478843631	0.1063344825532511	F	F	F
0.3749999913145174	0.5000000319229088	0.1063344825532511	F	F	F
0.8750000232374191	0.5000000319229088	0.1063344825532511	F	F	F
0.3749999913145174	0.7500000478843631	0.1063344825532511	F	F	F
0.8750000232374191	0.7500000478843631	0.1063344825532511	F	F	F
0.1249999753530631	0.1244328412915507	0.1659650172167275	T	T	T
0.6250000072759718	0.1244328412915507	0.1659650172167275	T	T	T
0.1249999753530631	0.3749999913145174	0.1727998729730587	T	T	T
0.6250000072759718	0.3749999913145174	0.1727998729730587	T	T	T
0.3749999913145174	0.1255671746699036	0.1659651095016983	T	T	T
0.8750000232374191	0.1255671746699036	0.1659651095016983	T	T	T
0.1249999753530631	0.6255670760821488	0.1659650633592165	T	T	T
0.6250000072759718	0.6255670760821488	0.1659650633592165	T	T	T
0.3749999913145174	0.3749999913145174	0.1715938468133729	T	T	T
0.8750000232374191	0.3749999913145174	0.1715938468133729	T	T	T
0.1249999753530631	0.8750000232374191	0.1715937083859131	T	T	T
0.6250000072759718	0.8750000232374191	0.1715937083859131	T	T	T
0.3749999913145174	0.6244328079591313	0.1659651095016983	T	T	T
0.8750000232374191	0.6244328079591313	0.1659651095016983	T	T	T
0.3749999913145174	0.8750000232374191	0.1727998729730587	T	T	T
0.8750000232374191	0.8750000232374191	0.1727998729730587	T	T	T
0.0000000000000000	0.1250003016297043	0.2266963731715421	T	T	T
0.5000000319229088	0.1250003016297043	0.2266963731715421	T	T	T
0.4991361818819158	0.3749999913145174	0.2373427367087331	T	T	T

0.9991362138048174	0.3749999913145174	0.2373427367087331	T	T	T
0.2499999507061261	0.1250003016297043	0.2266963731715421	T	T	T
0.7499999826290349	0.1250003016297043	0.2266963731715421	T	T	T
0.0000000000000000	0.6249996809993235	0.2266963731715421	T	T	T
0.5000000319229088	0.6249996809993235	0.2266963731715421	T	T	T
0.2508638007471191	0.3749999913145174	0.2373427367087331	T	T	T
0.7508638326700208	0.3749999913145174	0.2373427367087331	T	T	T
0.0008639805516495	0.8750000232374191	0.2373425059963026	T	T	T
0.5008639472192300	0.8750000232374191	0.2373425059963026	T	T	T
0.2499999507061261	0.6249996809993235	0.2266963731715421	T	T	T
0.7499999826290349	0.6249996809993235	0.2266963731715421	T	T	T
0.2491360354098049	0.8750000232374191	0.2373425059963026	T	T	T
0.7491360673327065	0.8750000232374191	0.2373425059963026	T	T	T
0.0081232443347119	0.0056880459792410	0.2964413869347240	T	T	T
0.5081232110022924	0.0056880459792410	0.2964413869347240	T	T	T
0.4918770486095099	0.2443120352375416	0.2964417560746142	T	T	T
0.9918770805324115	0.2443120352375416	0.2964417560746142	T	T	T
0.2418767716267425	0.0056880459792410	0.2964413869347240	T	T	T
0.7418768035496441	0.0056880459792410	0.2964413869347240	T	T	T
0.4918771138648381	0.5056879473914861	0.2964417560746142	T	T	T
0.9918770805324115	0.5056879473914861	0.2964417560746142	T	T	T
0.2581228687641968	0.2443121004928699	0.2964417560746142	T	T	T
0.7581228354317773	0.2443121004928699	0.2964417560746142	T	T	T
0.0081231790793836	0.7443120019051221	0.2964413869347240	T	T	T
0.5081231457469642	0.7443120019051221	0.2964413869347240	T	T	T
0.2581229340195250	0.5056878821361579	0.2964418022171031	T	T	T
0.7581229006871055	0.5056878821361579	0.2964418022171031	T	T	T
0.2418767716267425	0.7443120019051221	0.2964414330772129	T	T	T
0.7418768035496441	0.7443120019051221	0.2964414330772129	T	T	T
0.1249999753530631	0.0405486824083923	0.3650851641241957	T	T	T
0.6250000072759718	0.0405486824083923	0.3650851641241957	T	T	T
0.1249999753530631	0.1876281244951130	0.3309699477247889	T	T	T
0.6250000072759718	0.1876281244951130	0.3309699477247889	T	T	T
0.3749999913145174	0.0623711084024023	0.3309694862999280	T	T	T
0.8750000232374191	0.0623711084024023	0.3309694862999280	T	T	T
0.1249999753530631	0.5623719233892501	0.3309699477247889	T	T	T
0.6250000072759718	0.5623719233892501	0.3309699477247889	T	T	T
0.3749999913145174	0.2094503547231312	0.3650850256967431	T	T	T
0.8750000232374191	0.2094503547231312	0.3650850256967431	T	T	T
0.1249999753530631	0.7094513002206355	0.3650851641241957	T	T	T
0.6250000072759718	0.7094513002206355	0.3650851641241957	T	T	T
0.3749999913145174	0.5405496279059037	0.3650850718392249	T	T	T
0.8750000232374191	0.5405496279059037	0.3650850718392249	T	T	T
0.3749999913145174	0.6876288742266325	0.3309694862999280	T	T	T
0.8750000232374191	0.6876288742266325	0.3309694862999280	T	T	T
0.0000000000000000	0.1711557218778097	0.0044114523853764	F	F	F
0.5000000319229088	0.1711557218778097	0.0044114523853764	F	F	F
0.0000000000000000	0.3288443100450991	0.0044114523853764	F	F	F
0.5000000319229088	0.3288443100450991	0.0044114523853764	F	F	F
0.0000000000000000	0.4211557378392641	0.0044114523853764	F	F	F
0.5000000319229088	0.4211557378392641	0.0044114523853764	F	F	F
0.0000000000000000	0.0788442940836447	0.0044114523853764	F	F	F
0.5000000319229088	0.0788442940836447	0.0044114523853764	F	F	F
0.2500000159614544	0.0788442940836447	0.0044114523853764	F	F	F
0.7500000478843631	0.0788442940836447	0.0044114523853764	F	F	F
0.2500000159614544	0.1711557218778097	0.0044114523853764	F	F	F
0.7500000478843631	0.1711557218778097	0.0044114523853764	F	F	F
0.2500000159614544	0.3288443100450991	0.0044114523853764	F	F	F
0.7500000478843631	0.3288443100450991	0.0044114523853764	F	F	F
0.2500000159614544	0.4211557378392641	0.0044114523853764	F	F	F
0.7500000478843631	0.4211557378392641	0.0044114523853764	F	F	F
0.0000000000000000	0.6711557538007185	0.0044114523853764	F	F	F
0.5000000319229088	0.6711557538007185	0.0044114523853764	F	F	F
0.0000000000000000	0.8288443419680007	0.0044114523853764	F	F	F
0.5000000319229088	0.8288443419680007	0.0044114523853764	F	F	F
0.0000000000000000	0.9211557697621728	0.0044114523853764	F	F	F
0.5000000319229088	0.9211557697621728	0.0044114523853764	F	F	F
0.0000000000000000	0.5788443260065463	0.0044114523853764	F	F	F
0.5000000319229088	0.5788443260065463	0.0044114523853764	F	F	F
0.2500000159614544	0.5788443260065463	0.0044114523853764	F	F	F
0.7500000478843631	0.5788443260065463	0.0044114523853764	F	F	F
0.2500000159614544	0.6711557538007185	0.0044114523853764	F	F	F
0.7500000478843631	0.6711557538007185	0.0044114523853764	F	F	F
0.2500000159614544	0.8288443419680007	0.0044114523853764	F	F	F
0.7500000478843631	0.8288443419680007	0.0044114523853764	F	F	F
0.2500000159614544	0.9211557697621728	0.0044114523853764	F	F	F
0.7500000478843631	0.9211557697621728	0.0044114523853764	F	F	F

Single Adsorption structure **2**:

```
E = -745.44750 eV
   1.00000000000000
    15.3244190216000007    0.0000000000000000    0.0000000000000000
     0.0000000000000000   15.3244190216000007    0.0000000000000000
     0.0000000000000000    0.0000000000000000   21.6720008849999992
   Si   H    C
    96    44     8
Selective dynamics
Direct
  0.0000000000000000  0.0000000000000000  0.0438344851055064   F   F   F
  0.5000000319229088  0.0000000000000000  0.0438344851055064   F   F   F
  0.0000000000000000  0.2500000159614544  0.0438344851055064   F   F   F
  0.5000000319229088  0.2500000159614544  0.0438344851055064   F   F   F
  0.2500000159614544  0.0000000000000000  0.0438344851055064   F   F   F
  0.7500000478843631  0.0000000000000000  0.0438344851055064   F   F   F
  0.0000000000000000  0.5000000319229088  0.0438344851055064   F   F   F
  0.5000000319229088  0.5000000319229088  0.0438344851055064   F   F   F
  0.2500000159614544  0.2500000159614544  0.0438344851055064   F   F   F
  0.7500000478843631  0.2500000159614544  0.0438344851055064   F   F   F
  0.0000000000000000  0.7500000478843631  0.0438344851055064   F   F   F
  0.5000000319229088  0.7500000478843631  0.0438344851055064   F   F   F
  0.2500000159614544  0.5000000319229088  0.0438344851055064   F   F   F
  0.7500000478843631  0.5000000319229088  0.0438344851055064   F   F   F
  0.2500000159614544  0.7500000478843631  0.0438344851055064   F   F   F
  0.7500000478843631  0.7500000478843631  0.0438344851055064   F   F   F
  0.1249999753530631  0.0000000000000000  0.1063344825532511   F   F   F
  0.6250000072759718  0.0000000000000000  0.1063344825532511   F   F   F
  0.1249999753530631  0.2500000159614544  0.1063344825532511   F   F   F
  0.6250000072759718  0.2500000159614544  0.1063344825532511   F   F   F
  0.3749999913145174  0.0000000000000000  0.1063344825532511   F   F   F
  0.8750000232374191  0.0000000000000000  0.1063344825532511   F   F   F
  0.1249999753530631  0.5000000319229088  0.1063344825532511   F   F   F
  0.6250000072759718  0.5000000319229088  0.1063344825532511   F   F   F
  0.3749999913145174  0.2500000159614544  0.1063344825532511   F   F   F
  0.8750000232374191  0.2500000159614544  0.1063344825532511   F   F   F
  0.1249999753530631  0.7500000478843631  0.1063344825532511   F   F   F
  0.6250000072759718  0.7500000478843631  0.1063344825532511   F   F   F
  0.3749999913145174  0.5000000319229088  0.1063344825532511   F   F   F
  0.8750000232374191  0.5000000319229088  0.1063344825532511   F   F   F
  0.3749999913145174  0.7500000478843631  0.1063344825532511   F   F   F
  0.8750000232374191  0.7500000478843631  0.1063344825532511   F   F   F
  0.1250141681243089  0.1244087664280334  0.1658378464648347   T   T   T
  0.6249940954548070  0.1243861594611989  0.1659384944506375   T   T   T
  0.1249727485886840  0.3747935270566042  0.1729404309916690   T   T   T
  0.6248894821038682  0.3749398946166457  0.1728717039078044   T   T   T
  0.3750394233521298  0.1256040216356057  0.1659499465387764   T   T   T
  0.8749911854806076  0.1255944077105949  0.1659533817756433   T   T   T
  0.1250017356820791  0.6254380882998652  0.1665715976679410   T   T   T
  0.6249457356724329  0.6256187108329203  0.1661018381507085   T   T   T
  0.3749571973684508  0.3748129093654751  0.1713814153856061   T   T   T
  0.8746264804686171  0.3746801953036534  0.1711982384462448   T   T   T
  0.1249902514898502  0.8748490486785563  0.1713923136095730   T   T   T
  0.6250716038702487  0.8748601985965003  0.1717363293102363   T   T   T
  0.3741014344770064  0.6246426316619588  0.1662825430216507   T   T   T
  0.8757020407529661  0.6246797350754451  0.1662316910676253   T   T   T
  0.3757958429521973  0.8752734758743280  0.1722786255772141   T   T   T
  0.8743875357211067  0.8751907150315777  0.1723620856018005   T   T   T
  0.0000883432206187  0.1248191426464424  0.2265755674826295   T   T   T
  0.5000255146764417  0.1248387731215226  0.2267066118828138   T   T   T
  0.4989033985678012  0.3748235870509419  0.2372568094320880   T   T   T
  0.9980707678901689  0.3744116709942906  0.2367266377217644   T   T   T
  0.2499512185161215  0.1250763114334225  0.2265635510259217   T   T   T
  0.7499773188979918  0.1247900781353149  0.2266763484331372   T   T   T
 -0.0000034281179504  0.6224517202417723  0.2274550454780087   T   T   T
  0.4996922169332091  0.6245622801739884  0.2266165570016008   T   T   T
  0.2514819478433908  0.3750391971858278  0.2372101937144462   T   T   T
  0.7508431109441929  0.3748242102015570  0.2372321379014447   T   T   T
  0.0000947954595898  0.8762909011941261  0.2364164909642981   T   T   T
  0.5009565206176302  0.8746231387181165  0.2376084776925406   T   T   T
  0.2498467713210204  0.6224359496818661  0.2276417028281749   T   T   T
  0.7501857955597084  0.6245046283354655  0.2266249316642029   T   T   T
  0.2500943168449558  0.8766961854640711  0.2361679060745762   T   T   T
  0.7491809527826147  0.8745314129621681  0.2376326758526232   T   T   T
  0.0081216886169534  0.0058383737933860  0.2964339802990478   T   T   T
  0.4917247916109967  0.2442425876153243  0.2964609436897268   T   T   T
  0.2419256291333678  0.0060447559818099  0.2963595950684692   T   T   T
```

0.4918379333683401	0.5052407286512600	0.2965908191028861	T	T	T
0.2580063570265876	0.2443901989369928	0.2961774517483940	T	T	T
0.0016761609952838	0.7458618233465966	0.2950779474507273	T	T	T
0.2556523162761969	0.5046869614754550	0.2968170442504126	T	T	T
0.2490504829413601	0.7464689346202713	0.2947391558187120	T	T	T
0.5087670343612398	0.7437391466396032	0.2968514661594585	T	T	T
0.5081691812692299	0.0055094326855145	0.2965097004575461	T	T	T
0.9917923117615097	0.2441969300916325	0.2961270837825408	T	T	T
0.7418795780332533	0.0054633930091081	0.2964653090527107	T	T	T
0.9930222058687772	0.5039342158047048	0.2959136485362141	T	T	T
0.7580817570983095	0.2442088625878395	0.2963719514847623	T	T	T
0.7578162074156169	0.5052202499543179	0.2966028548151242	T	T	T
0.7415663705262319	0.7436069615529917	0.2968471809602162	T	T	T
0.1250268589913670	0.0401990279093790	0.3653079090408162	T	T	T
0.1249246206466143	0.1875735031509044	0.3307473968154829	T	T	T
0.3750991839199998	0.0624352399107506	0.3309246424915939	T	T	T
0.1235501120233963	0.5414500300446848	0.3452409205576938	T	T	T
0.3746905618826580	0.2094043840218014	0.3650106301573143	T	T	T
0.1257361770720553	0.6942670753791738	0.3466631237783302	T	T	T
0.3741648012422614	0.5376785752039666	0.3649125394608479	T	T	T
0.3775812859518192	0.6841671860090766	0.3317332964495034	T	T	T
0.6250412610879512	0.0406575846098195	0.3650710091858343	T	T	T
0.6249210942059916	0.1876940991786624	0.3308882395601814	T	T	T
0.8749316221522380	0.0623582810806081	0.3309074538039358	T	T	T
0.6247976059287853	0.5618524179771439	0.3309040876335029	T	T	T
0.8750942849964356	0.2093203637825420	0.3649320648730933	T	T	T
0.6251083099885952	0.7088809349719984	0.3654921899976730	T	T	T
0.8757353090260381	0.5372777685334869	0.3648495432840176	T	T	T
0.8727505109206065	0.6836719439886089	0.3316757311531301	T	T	T
0.0000000000000000	0.1711557218778097	0.0044114523853764	F	F	F
0.5000000319229088	0.1711557218778097	0.0044114523853764	F	F	F
0.0000000000000000	0.3288443100450991	0.0044114523853764	F	F	F
0.5000000319229088	0.3288443100450991	0.0044114523853764	F	F	F
0.0000000000000000	0.4211557378392641	0.0044114523853764	F	F	F
0.5000000319229088	0.4211557378392641	0.0044114523853764	F	F	F
0.0000000000000000	0.0788442940836447	0.0044114523853764	F	F	F
0.5000000319229088	0.0788442940836447	0.0044114523853764	F	F	F
0.2500000159614544	0.0788442940836447	0.0044114523853764	F	F	F
0.7500000478843631	0.0788442940836447	0.0044114523853764	F	F	F
0.2500000159614544	0.1711557218778097	0.0044114523853764	F	F	F
0.7500000478843631	0.1711557218778097	0.0044114523853764	F	F	F
0.2500000159614544	0.3288443100450991	0.0044114523853764	F	F	F
0.7500000478843631	0.3288443100450991	0.0044114523853764	F	F	F
0.2500000159614544	0.4211557378392641	0.0044114523853764	F	F	F
0.7500000478843631	0.4211557378392641	0.0044114523853764	F	F	F
0.0000000000000000	0.6711557538007185	0.0044114523853764	F	F	F
0.5000000319229088	0.6711557538007185	0.0044114523853764	F	F	F
0.0000000000000000	0.8288443419680007	0.0044114523853764	F	F	F
0.5000000319229088	0.8288443419680007	0.0044114523853764	F	F	F
0.0000000000000000	0.9211557697621728	0.0044114523853764	F	F	F
0.5000000319229088	0.9211557697621728	0.0044114523853764	F	F	F
0.0000000000000000	0.5788443260065463	0.0044114523853764	F	F	F
0.5000000319229088	0.5788443260065463	0.0044114523853764	F	F	F
0.2500000159614544	0.5788443260065463	0.0044114523853764	F	F	F
0.7500000478843631	0.5788443260065463	0.0044114523853764	F	F	F
0.2500000159614544	0.6711557538007185	0.0044114523853764	F	F	F
0.7500000478843631	0.6711557538007185	0.0044114523853764	F	F	F
0.2500000159614544	0.8288443419680007	0.0044114523853764	F	F	F
0.7500000478843631	0.8288443419680007	0.0044114523853764	F	F	F
0.2500000159614544	0.9211557697621728	0.0044114523853764	F	F	F
0.7500000478843631	0.9211557697621728	0.0044114523853764	F	F	F
0.3233749149183499	0.5134713628696981	0.5569096345672125	T	T	T
0.2240449270098963	0.5314538285131194	0.5958768085144072	T	T	T
0.2066240501883638	0.4141713614618823	0.5227763590281519	T	T	T
0.2524736696040594	0.4749640296389854	0.4617744259712114	T	T	T
0.0825780629234305	0.5362472145269855	0.5228616160703173	T	T	T
0.1776888679383492	0.7798990306106393	0.4690562198266148	T	T	T
0.3125937526620459	0.6653147226469018	0.5661946309974262	T	T	T
0.2868517937369895	0.6454116363174534	0.4884396985282369	T	T	T
0.1280438769387581	0.6466371727357706	0.5684065606359434	T	T	T
0.0725231590680319	0.7545570764370017	0.4947469895438074	T	T	T
0.1872593919863353	0.7436751949924839	0.5758756573402036	T	T	T
0.0821902301958644	0.4521599713725961	0.4675140311262938	T	T	T
0.1273083466827175	0.6631221729326292	0.4315221706414832	T	T	T
0.2566692089631503	0.5389941672261868	0.5507111791511895	T	T	T
0.2110612384630260	0.4804663804876852	0.5033282871624850	T	T	T
0.1368449897925336	0.7248404395483864	0.4849600364369813	T	T	T

0.1761945883416488	0.6880029183631021	0.5446905021073871	T	T	T
0.2623639913395801	0.6368471624929279	0.5359610999988265	T	T	T
0.1188915215501037	0.5098953648062663	0.4831757873367582	T	T	T
0.1214143304674174	0.5740589207588955	0.4305509907177211	T	T	T

Double adsorption structure **3**

```
E = -863.76248 eV
   1.00000000000000
    15.3244190216000007    0.0000000000000000    0.0000000000000000
     0.0000000000000000   15.3244190216000007    0.0000000000000000
     0.0000000000000000    0.0000000000000000   21.6720008849999992
   Si   H    C
    96    56    16
Selective dynamics
Direct
  0.0000000000000000  0.1250000000000000  0.0438344851055064   F   F   F
  0.5000000319229088  0.1250000000000000  0.0438344851055064   F   F   F
  0.0000000000000000  0.3750000159614544  0.0438344851055064   F   F   F
  0.5000000319229088  0.3750000159614544  0.0438344851055064   F   F   F
  0.2500000159614544  0.1250000000000000  0.0438344851055064   F   F   F
  0.7500000478843631  0.1250000000000000  0.0438344851055064   F   F   F
  0.0000000000000000  0.6250000319229088  0.0438344851055064   F   F   F
  0.5000000319229088  0.6250000319229088  0.0438344851055064   F   F   F
  0.2500000159614544  0.3750000159614544  0.0438344851055064   F   F   F
  0.7500000478843631  0.3750000159614544  0.0438344851055064   F   F   F
  0.0000000000000000  0.8750000478843631  0.0438344851055064   F   F   F
  0.5000000319229088  0.8750000478843631  0.0438344851055064   F   F   F
  0.2500000159614544  0.6250000319229088  0.0438344851055064   F   F   F
  0.7500000478843631  0.6250000319229088  0.0438344851055064   F   F   F
  0.2500000159614544  0.8750000478843631  0.0438344851055064   F   F   F
  0.7500000478843631  0.8750000478843631  0.0438344851055064   F   F   F
  0.1249999753530631  0.1250000000000000  0.1063344825532511   F   F   F
  0.6250000072759718  0.1250000000000000  0.1063344825532511   F   F   F
  0.1249999753530631  0.3750000159614544  0.1063344825532511   F   F   F
  0.6250000072759718  0.3750000159614544  0.1063344825532511   F   F   F
  0.3749999913145174  0.1250000000000000  0.1063344825532511   F   F   F
  0.8750000232374191  0.1250000000000000  0.1063344825532511   F   F   F
  0.1249999753530631  0.6250000319229088  0.1063344825532511   F   F   F
  0.6250000072759718  0.6250000319229088  0.1063344825532511   F   F   F
  0.3749999913145174  0.3750000159614544  0.1063344825532511   F   F   F
  0.8750000232374191  0.3750000159614544  0.1063344825532511   F   F   F
  0.1249999753530631  0.8750000478843631  0.1063344825532511   F   F   F
  0.6250000072759718  0.8750000478843631  0.1063344825532511   F   F   F
  0.3749999913145174  0.6250000319229088  0.1063344825532511   F   F   F
  0.8750000232374191  0.6250000319229088  0.1063344825532511   F   F   F
  0.3749999913145174  0.8750000478843631  0.1063344825532511   F   F   F
  0.8750000232374191  0.8750000478843631  0.1063344825532511   F   F   F
  0.6250458768319953  0.7494173720198544  0.1658489351528889   T   T   T
  0.1249703632303770  0.7493765308334530  0.1659344965407540   T   T   T
  0.6255392742957876  0.9996003507886853  0.1724503035874567   T   T   T
  0.1239405898041669  0.9995824338253939  0.1721594247685772   T   T   T
  0.8750307041554841  0.7506249377329544  0.1659397312018406   T   T   T
  0.3749807138328354  0.7505663762016529  0.1658451738778831   T   T   T
  0.6240361583417406  0.2502072305855763  0.1668902700989136   T   T   T
  0.1260466459181835  0.2503979679248243  0.1663718628383971   T   T   T
  0.8747998853251747  0.9999709880546463  0.1715958322135345   T   T   T
  0.3747452040469289  0.9998706941468382  0.1711437875629161   T   T   T
  0.6253217351653803  0.5001421866738176  0.1711122650045757   T   T   T
  0.1252297309133124  0.5000426385196347  0.1716048784783756   T   T   T
  0.8741699195832977  0.2495987210815171  0.1663640308009686   T   T   T
  0.3761349159551940  0.2497814974163569  0.1669430656788945   T   T   T
  0.8761902147114108  0.5004084130365090  0.1721638392056912   T   T   T
  0.3745177876200778  0.5004027423886259  0.1724687688612291   T   T   T
  0.5000125131175231  0.7499900329374561  0.2264822868093001   T   T   T
  0.0000019290740086  0.7499869624188049  0.2267199972108667   T   T   T
  0.9988627926052428  1.0002142189517824  0.2375008459901132   T   T   T
  0.4991385849814439  0.9982818181922838  0.2360887772937388   T   T   T
  0.7499799997725957  0.7503638725264855  0.2265866324870788   T   T   T
  0.2500368924507912  0.7496345227466691  0.2265628916638656   T   T   T
  0.5001372601363635  0.2499975255365502  0.2289757506408274   T   T   T
  1.0001121822869921  0.2500299623770910  0.2266043946887445   T   T   T
  0.7512298193087241  0.0005978476260311  0.2375415787487571   T   T   T
  0.2495513448716639  0.9978325761904357  0.2357878417959967   T   T   T
  0.5009825242290306  0.5017362886808869  0.2360825220205406   T   T   T
  0.0012396996831933  0.4997571382878557  0.2375639943972245   T   T   T
  0.7495626520178742  0.2477881083324258  0.2273515075103968   T   T   T
  0.2506150890172756  0.2521608752560806  0.2274379193134108   T   T   T
  0.7505558725883835  0.5021903579474382  0.2357348442946910   T   T   T
  0.2488008622397888  0.4994263545123018  0.2375361434854856   T   T   T
  0.5082266330326791  0.6310142453004228  0.2961963544540651   T   T   T
  0.9917488426130012  0.8694001485480275  0.2965263652420080   T   T   T
  0.7422348200032091  0.6312031550509842  0.2962503708771026   T   T   T
```

0.9911082975288728	0.1307802011581458	0.2970828315904844	T	T	T
0.7579182761780215	0.8696691737283806	0.2962406014373472	T	T	T
0.5009459262936545	0.3727375072054019	0.2953423080239831	T	T	T
0.7556076778302020	0.1306819927430758	0.2971015138592375	T	T	T
0.7499432473349178	0.3721176707773941	0.2940612748650562	T	T	T
0.0091318215999888	0.3692018772040694	0.2971920551093939	T	T	T
0.0083263861388128	0.6305949775690873	0.2965439262909874	T	T	T
0.4918862935687798	0.8689752467926163	0.2961804950443742	T	T	T
0.2421509914761094	0.6303445482085022	0.2962178008301141	T	T	T
0.4992769402578785	0.1272518396994526	0.2953585285979003	T	T	T
0.2578869595471813	0.8687702696634590	0.2962419292556190	T	T	T
0.2502093053001228	0.1278413566896734	0.2942189928057231	T	T	T
0.2444364555653532	0.3693606979232508	0.2971370528780048	T	T	T
0.6251269333355091	0.6649919008464951	0.3651499765240679	T	T	T
0.6250382684807287	0.8123488750916132	0.3308449999979428	T	T	T
0.8753076488453468	0.6876310950619584	0.3309602103756353	T	T	T
0.6257873384377641	0.1708403644060582	0.3470085844657207	T	T	T
0.8746805009246614	0.8346286220721398	0.3650457188992052	T	T	T
0.6260724499479050	0.3236149929372902	0.3465497538497341	T	T	T
0.8737580313382282	0.1632961889702515	0.3652744078704793	T	T	T
0.8778677806166547	0.3097898755351714	0.3316639412740707	T	T	T
0.1254071464000936	0.6654007486360687	0.3650270527322490	T	T	T
0.1247828977757863	0.8123896598088938	0.3309410776571327	T	T	T
0.3750593756111557	0.6876260190730411	0.3308199194686139	T	T	T
0.1222775801927535	0.1902512570019632	0.3317193764653707	T	T	T
0.3749946852120969	0.8349493626777169	0.3651510513887321	T	T	T
0.1264563401257922	0.3366980357407679	0.3654945757401702	T	T	T
0.3742245910731792	0.1764614551331914	0.3465217384536162	T	T	T
0.3743457883563010	0.3292192970880655	0.3468796670911504	T	T	T
0.0000000000000000	0.2961557200000016	0.0044114523853764	F	F	F
0.5000000319229088	0.2961557200000016	0.0044114523853764	F	F	F
0.0000000000000000	0.4538443100000009	0.0044114523853764	F	F	F
0.5000000319229088	0.4538443100000009	0.0044114523853764	F	F	F
0.0000000000000000	0.5461557400000032	0.0044114523853764	F	F	F
0.5000000319229088	0.5461557400000032	0.0044114523853764	F	F	F
0.0000000000000000	0.2038442899999993	0.0044114523853764	F	F	F
0.5000000319229088	0.2038442899999993	0.0044114523853764	F	F	F
0.2500000159614544	0.2038442899999993	0.0044114523853764	F	F	F
0.7500000478843631	0.2038442899999993	0.0044114523853764	F	F	F
0.2500000159614544	0.2961557200000016	0.0044114523853764	F	F	F
0.7500000478843631	0.2961557200000016	0.0044114523853764	F	F	F
0.2500000159614544	0.4538443100000009	0.0044114523853764	F	F	F
0.7500000478843631	0.4538443100000009	0.0044114523853764	F	F	F
0.2500000159614544	0.5461557400000032	0.0044114523853764	F	F	F
0.7500000478843631	0.5461557400000032	0.0044114523853764	F	F	F
0.0000000000000000	0.7961557499999969	0.0044114523853764	F	F	F
0.5000000319229088	0.7961557499999969	0.0044114523853764	F	F	F
0.0000000000000000	0.9538443400000034	0.0044114523853764	F	F	F
0.5000000319229088	0.9538443400000034	0.0044114523853764	F	F	F
0.0000000000000000	0.0461557699999986	0.0044114523853764	F	F	F
0.5000000319229088	0.0461557699999986	0.0044114523853764	F	F	F
0.0000000000000000	0.7038443300000026	0.0044114523853764	F	F	F
0.5000000319229088	0.7038443300000026	0.0044114523853764	F	F	F
0.2500000159614544	0.7038443300000026	0.0044114523853764	F	F	F
0.7500000478843631	0.7038443300000026	0.0044114523853764	F	F	F
0.2500000159614544	0.7961557499999969	0.0044114523853764	F	F	F
0.7500000478843631	0.7961557499999969	0.0044114523853764	F	F	F
0.2500000159614544	0.9538443400000034	0.0044114523853764	F	F	F
0.7500000478843631	0.9538443400000034	0.0044114523853764	F	F	F
0.2500000159614544	0.0461557699999986	0.0044114523853764	F	F	F
0.7500000478843631	0.0461557699999986	0.0044114523853764	F	F	F
0.8244994982063770	0.1500018900873065	0.5638546272503271	T	T	T
0.7229733388023399	0.1681046194286781	0.5998486555364752	T	T	T
0.7120824952563161	0.0486730596208778	0.5273021082208128	T	T	T
0.7584761069839119	0.1101136684499080	0.4668020616157146	T	T	T
0.5855983788331198	0.1684964075481810	0.5247107084912997	T	T	T
0.6841223006510658	0.4100160637452298	0.4670040436856133	T	T	T
0.8121046037952412	0.3019600781189986	0.5708635042570859	T	T	T
0.7908425810812852	0.2797866770943236	0.4927177665222635	T	T	T
0.6277507787091907	0.2823941638239789	0.5684315216760577	T	T	T
0.5773547367736417	0.3898748284839724	0.4914412249478390	T	T	T
0.6861077372993075	0.3800462720334964	0.5755046811415305	T	T	T
0.5884618337041040	0.0825481979951307	0.4707563356035426	T	T	T
0.1835301476316507	0.3484015109698054	0.5692607815884186	T	T	T
0.2874135753012334	0.3313338486966880	0.6019822923924369	T	T	T
0.2919231626620435	0.4507447164066434	0.5288176544112914	T	T	T
0.2433711429119058	0.3879936225275135	0.4698604181314135	T	T	T

0.4198708431071222	0.3325688970502733	0.5235270306031720	T	T	T
0.3199156723638040	0.0899740137483286	0.4681584765079170	T	T	T
0.1976094606363262	0.1965585073830042	0.5758716043883501	T	T	T
0.2140995128124939	0.2188215119619295	0.4971473648055827	T	T	T
0.3815241255665119	0.2178049527321546	0.5679500506025511	T	T	T
0.4279102349346131	0.1101925639201137	0.4896617120043708	T	T	T
0.3245089660599750	0.1196517828164799	0.5768573712321828	T	T	T
0.4132774948337991	0.4182731759093807	0.4695489084759625	T	T	T
0.6305759869315409	0.2934345482889182	0.4317054075405208	T	T	T
0.7579930653897868	0.1748415376147979	0.5555136362417482	T	T	T
0.7155253052270361	0.1148883839988184	0.5076103303686560	T	T	T
0.6408538537919163	0.3573175827657999	0.4837728035506272	T	T	T
0.6769391477903995	0.3230982624346422	0.5451687506932126	T	T	T
0.7638143342336584	0.2722839632167255	0.5396426908714633	T	T	T
0.6233381882399909	0.1417534546298577	0.4857827719966476	T	T	T
0.6248911954218905	0.2043554060765742	0.4321285633656421	T	T	T
0.3715200103011477	0.2067446086779491	0.4316712345041597	T	T	T
0.2496527388625317	0.3241479782897316	0.5588200788025434	T	T	T
0.2884845015214210	0.3842973402807727	0.5095305333758527	T	T	T
0.3639798535841298	0.1426805800855374	0.4838443361048327	T	T	T
0.3313240271919751	0.1766174835938419	0.5462385239112880	T	T	T
0.2437358349407047	0.2266287818439633	0.5432456692412351	T	T	T
0.3799371058066418	0.3586566851663160	0.4854838440886210	T	T	T
0.3767650485179009	0.2958541934923828	0.4319870396484356	T	T	T
